# Study on Centroid Height Prediction of Non-Rigid Vehicle Based on Deep Learning Combined Model

**DOI:** 10.3390/s25185692

**Published:** 2025-09-12

**Authors:** Guoqiang Pang, Zhiquan Xiao, Zhanwen Cai, Pei Wang

**Affiliations:** School of Mechanical Engineering and Automation, Wuhan Textile University, Wuhan 430200, China; guoqiang_2000@163.com (G.P.); zhanwen_2000@163.com (Z.C.); wangpei_wtu@163.com (P.W.)

**Keywords:** height of center of gravity (*Z_CG_*), tilt-table, unlocked suspension systems, variable sprung mass conditions, CNN–LSTM–Attention model

## Abstract

The height of the center of gravity (*Z_CG_*) is a critical parameter for evaluating vehicle safety and performance. Systematic errors arise in *Z_CG_* measurement via the tilt-table test method due to unlocked suspension systems and variable sprung mass conditions, which compromise accuracy. To address this limitation, a CNN–LSTM–Attention model integrating convolutional neural networks (CNNs), long short-term memory networks (LSTMs), and an attention mechanism is proposed. The CNN extracts spatial correlations among vehicle load transfer, suspension stiffness, and tilt angles. The LSTM captures temporal dependencies in tilt angle sequences, while the attention mechanism amplifies critical load-transfer features near the 0° region. Simulations of vehicles with unlocked suspension and variable sprung mass were conducted in Adams using tilt-table protocols. The CNN–LSTM–Attention model was trained on simulation data and validated with real-world tilt-test data under identical suspension conditions. Results demonstrate that the CNN–LSTM–Attention model achieves at least a 6.9% improvement in computational speed and at least a 0.1% reduction in prediction error compared to CNN, CNN-LSTM, and Transformer baselines. The CNN–LSTM–Attention model demonstrates valid predictive capability for *Z_CG_* at 0° tilt angle. This novel approach provides a robust solution for the tilt-table test method *Z_CG_* measurement, enhancing practical accuracy in vehicle dynamics parameter quantification.

## 1. Introduction

*Z_CG_* is a critical parameter for evaluating vehicle performance and safety, with its accuracy directly impacting engineering design decisions [1]. Current *Z_CG_* measurement methods include the axle-lift method, stabilized pendulum method, and tilt-table test [2]. While suitable for small vehicles, the axle-lift method and stabilized pendulum method exhibit limitations when applied to large vehicles such as semi-trailers or full trailers. In the conventional tilt-table method, the vehicle with locked suspension is mounted on a platform, and load transfer variations are recorded at left/right tilt angles of 6–12°. The *Z_CG_* at 0° is then approximated by arithmetic averaging of *Z_CG_* values within this angular range, introducing systematic errors due to angle-dependent extrapolation. Suspension lock procedures present operational complexities and safety risks, motivating research into unlocked-suspension measurement approaches [3]. When measuring *Z_CG_* without locking the suspension, tilt-induced deformation occurs, violating the rigid-body assumption essential for conventional methods [4]. Consequently, suspension deformation induces coordinate shifts in the longitudinal position of the center of gravity (*X_CG_*), the lateral position of the center of gravity (*Y_CG_*), and *Z_CG_* dimensions. Furthermore, a coupling effect between *Y_CG_* and *Z_CG_* makes it challenging to measure either parameter independently with high precision. Aftermarket functional components retrofitted to factory vehicles create variable sprung mass conditions, introducing additional uncertainties. Component heterogeneity and positional variability amplify suspension deformation magnitudes, thereby exacerbating fluctuations in *X_CG_*, *Y_CG_*, and *Z_CG_* coordinates. Collectively, three factors compromise *Z_CG_* measurement accuracy:

(1) angle-dependent extrapolation via arithmetic averaging within 6–12° tilt intervals,
(2) *Y_CG_*-*Z_CG_* coupling under unlocked suspension impeding independent measurement,
(3) variable sprung mass exacerbating deformation-induced coordinate shifts.

Conventional approaches to these issues include time-domain analysis [5], frequency-domain analysis [6], and wavelet transforms [7]. Recent studies have integrated conventional techniques with machine learning algorithms, such as combining fast Fourier transform (FFT) with support vector machines (SVMs) [8], short-time Fourier transform (STFT) with CNN [9], or wavelet transforms with LSTM [10]. As a pivotal branch of machine learning, deep learning (DL) has been widely applied across multiple domains [11] due to its strengths in feature extraction, time-series modeling, robustness, and generalization capability. For instance, Hwang et al. implemented a CNN–LSTM–Attention model for monitoring human fatigue [12]. Wang Xingfen et al. proposed a CNN–LSTM–Attention-based approach to improve the accuracy of iron ore futures price prediction [13]. Yang Yong et al. developed an intelligent diagnostic approach using a CNN–LSTM–Attention model, which enhances the accuracy and reliability of track circuit fault diagnosis [14]. Xia K. et al. developed a CNN–BiLSTM–Attention-based model for predicting the aging status of automotive wiring harnesses [15]. Chen Xing et al. developed a CNN–LSTM–Attention-based model for predicting monthly domestic water demand, effectively forecasting water consumption patterns [16]. She Chengxi et al. developed an intelligent fault diagnosis and early warning method for production lines using a CNN–LSTM–Attention model, enabling effective fault prediction through data mining [17]. Zhu Anfeng et al. developed a condition monitoring and health evaluation system for wind turbines using a CNN–LSTM–Attention model [18].

Given that the tilt-table method does not lock the suspension system and that variations in sprung mass can affect the measurement of the *Z_CG_*, this study proposes a CNN–LSTM–Attention model—integrating CNN, LSTM, and attention mechanisms—to predict the 0° *Z_CG_* of a typical two-axle vehicle, leveraging the advantages of deep learning and its successful application cases.

## 2. Tilt-Table

### 2.1. Tilt-Table Method

**Figure 1:**
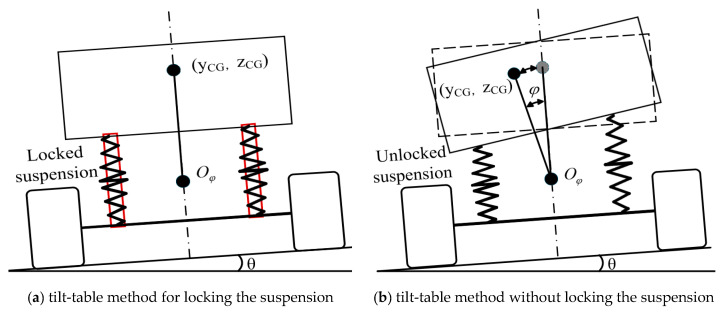
tilt-table method.

As illustrated in Figure 1a, the tilt-table method is based on a simplified vehicle model assuming non-independent suspension, wherein the suspension and tires remain undeformed during tilting, and fluid movement does not affect the measurement. The tested vehicle is mounted on the tilt-table and tilted to ±12°, respectively. Load transfer data are collected at each tilt angle, and the arithmetic average of the *Z_CG_* between 6° and 12° is taken as the estimated *Z_CG_* at 0°. Using this approach, the maximum measurement error of *Z_CG_* can reach up to 0.31% [19].

### 2.2. Unlocked Suspension Systems

As illustrated in Figure 1b, the tilt-table method assumes the vehicle to be a rigid body; however, this assumption is invalid when the suspension is not locked. With increasing tilt angle, the suspension deformation becomes more pronounced, resulting in a significant change in *Y_CG_*. As shown in Table 1, the maximum error introduced in *Y_CG_* due to this effect can reach up to 0.65%.

Under unlocked suspension conditions, the measurement error in *Z_CG_* is exponentially amplified by *Y_CG_* variations due to interdependent deformation effects.

### 2.3. Variable Sprung Mass

With increasing functional requirements, retrofitting aftermarket customized components to factory vehicles creates variable sprung mass conditions. This alters the weight distribution borne by the suspension system. Given the component heterogeneity and positional variability, the center-of-gravity position of the sprung mass shifts, consequently modifying the overall vehicle center-of-gravity coordinates. During tilt-table testing, unlocked suspension states (Section 2.2) combined with variable sprung mass dynamics cause the vehicle center-of-gravity position coordinates to vary with tilt angles, thereby amplifying measurement errors in *Z_CG_*.

Conventional approaches, constrained by rigid-body assumptions and static physical modeling of vehicles, struggle to resolve the aforementioned issues. In contrast, deep learning leverages end-to-end automatic feature extraction mechanisms through multi-layer nonlinear network architectures, directly processing raw sensor data (such as wheel load time-series signals, body tilt angles) to extract multi-level and multi-scale features of suspension dynamics during tilt-table testing. This methodology not only reveals correlations among tilt angles, suspension deformations, and wheel load variations under variable sprung mass conditions but also captures spatiotemporal dependencies in suspension response sequences. Consequently, it offers a novel solution for *Z_CG_* measurement during tilt-table testing with unlocked suspension states and variable sprung mass configurations.

## 3. *Z_CG_* Prediction Model with Unlocked Suspension States and Variable Sprung Mass Configurations

### 3.1. CNN Model

Owing to its exceptional feature extraction capabilities, the CNN model has gained widespread recognition in deep learning applications [20,21,22]. Through the synergistic interaction of convolutional operations and nonlinear activation functions, this architecture enables the extraction and representation of load transfer characteristics during vehicle roll maneuvers.

**Figure 2:**
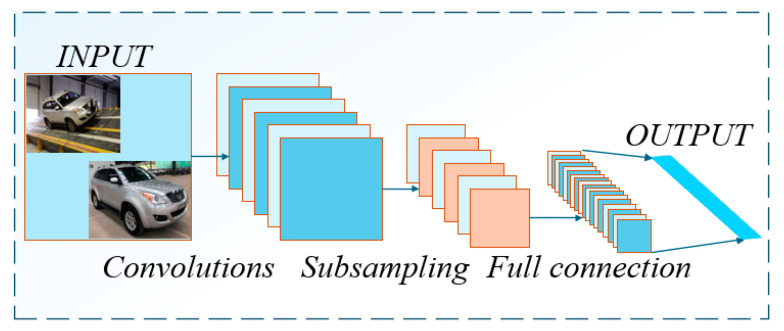
Structure of the CNN model.

As illustrated in Figure 2, a CNN primarily consists of convolutional layers, pooling layers, and fully connected layers. By alternately stacking these layers, CNNs are capable of progressively extracting informative features from raw sequence data [23]. For instance, when input data are presented, feature extraction is performed by the convolutional layer according to Equation (1).

(1) $$h_{i}=f(W_{i}⨀X+b_{i})$$

where $h_{i}$ denotes the feature map output from layer i; f(·) represents the activation function; W corresponds to the convolution kernel weight matrix; and $b_{i}$ is the bias vector.

CNNs are employed to extract features of wheel load transfer during vehicle roll on a tilt-table test platform, under conditions of unlocked suspension and variable sprung mass. However, given the temporal characteristics inherent in wheel load transfer during the roll process, CNN and LSTM are integrated to enhance prediction performance.

### 3.2. LSTM Model

**Figure 3:**
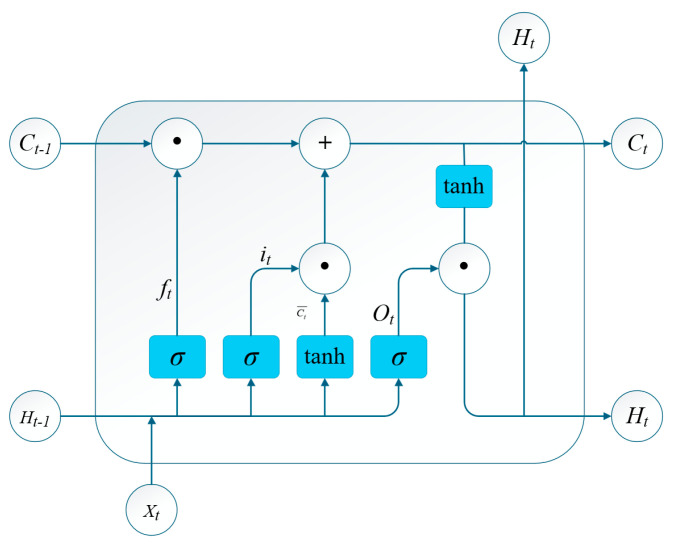
Structure of the LSTM model.

LSTM effectively integrates state information across sequential positions. Its unique architecture mitigates the vanishing gradient problem commonly encountered in recurrent neural network training [24]. As illustrated in Figure 3, LSTM regulates historical information through gating structures: the input gate $i_{t}$, forget gate $f_{t}$, and output gate $O_{t}$. These mechanisms enable LSTM to capture long-term dependencies during wheel load transfer while enhancing feature extraction capabilities [25].

The forget gate $f_{t}$ is computed as shown in Equation (2):

(2) $$f_{t}=\sigma (W_{f}\cdot [H_{t-1},X_{t}]+b_{f})$$

where σ denotes the sigmoid activation function; $W_{f}$ is the forget gate weight matrix; $H_{t-1}$ is the hidden state at time t−1; $X_{t}$ is the input vector at time t; and $b_{f}$ is the bias vector.

The input gate regulates information flow into the cell state $C_{t}$ through Equations (3)–(5):

(3) $$i_{t}=\sigma (W_{i}\cdot [H_{t-1},X_{t}]+b_{i})$$

(4) $$\bar{C_{t}}=tanh(W_{c}\cdot [H_{t-1},X_{t}]+b_{c})$$

(5) $$C_{t}=f_{t}C_{t-1}+i_{t}\bar{C_{t}}$$

Here, $i_{t}$ is the input gate; $W_{i}$ is the input gate weight matrix; $b_{i}$ and $b_{c}$ are bias vectors for the input gate and candidate cell state, respectively; $\bar{C_{t}}$ is the candidate cell state at t; tanh is the hyperbolic tangent activation; and $C_{t}$ represents the updated cell state.

The output gate governs the final output through Equations (6) and (7):

(6) $$O_{t}=\sigma (W_{o}\cdot {[H}_{t-1},X_{t}]+b_{o})$$

(7) $$H_{t}=O_{t}\cdot tanh(C_{t})$$

where $O_{t}$ is the output gate; $W_{o}$ is the output gate weight matrix; $b_{o}$ is the bias vector; and $h_{t}$ is the hidden state output at time t.

The CNN-LSTM model is capable of extracting local features and capturing roll sequence dependencies. However, in processing extended sequences, it is not sufficient to accentuate critical patterns such as minimal load transfer near 0° roll angle and suspension stiffness–roll angle interdependencies. To enhance focus adaptability, an attention mechanism dynamically weights salient features, thereby improving model prediction accuracy.

### 3.3. Attention Mechanism

The attention mechanism is a computational model that simulates selective focus on critical information in human cognition. Its core principle dynamically weights input components by computing inter-element correlations, thereby enhancing the model’s focus on salient features [26]. The computational procedure is defined by Equations (8)–(10):

(8) $$E_{t}=tanh\cdot H_{t}$$

(9) $$a_{t}=softmax(w_{a}^{T}\cdot E_{t})$$

(10) $$A_{t}=H_{t}\cdot a_{t}^{T}$$

where $H_{t}$ is the LSTM output matrix at time t; $E_{t}$ denotes the tanh-transformed feature representation; $w_{a}^{T}$ represents the transposed weight vector; $a_{t}$ is the softmax-normalized attention vector; and $A_{t}$ is the attention-weighted output at t [27].

### 3.4. Construction of the CNN–LSTM–Attention Model

In this study, the input for each sample is a four-channel spatiotemporal tensor with dimensions of 120 × 4 × 2 × 2, as denoted by Equations (11)–(16).

(11) $$I^{\left(i\right)}=\left[I^{\left(i,1\right)},I^{\left(i,2\right)},\ldots I^{\left(i,120\right)}\right]\in R^{120\times 4\times 2\times 2}$$

(12) $$I^{\left(i,t\right)}=\left[M^{\left(i,t\right)},M_{LR}^{\left(i,t\right)},M_{Front}^{\left(i,t\right)},M_{Diag}^{\left(i,t\right)}\right]$$

(13) $$M^{\left(i,t\right)}=\left[\begin{array}{cc} F_{fl}^{\left(i,t\right)} & F_{fr}^{\left(i,t\right)}\\ F_{rl}^{\left(i,t\right)} & F_{rr}^{\left(i,t\right)} \end{array}\right]\in R^{2\times 2}$$

(14) $$M_{LR}^{\left(i,t\right)}=\left[\begin{array}{cc} F_{fl}-F_{fr} & F_{fl}-F_{fr}\\ F_{rl}-F_{rr} & F_{rl}-F_{rr} \end{array}\right]$$

(15) $$M_{Front}^{\left(i,t\right)}=\left[\begin{array}{cc} F_{fl}+F_{fr} & F_{fl}+F_{fr}\\ F_{rl}+F_{rr} & F_{rl}+F_{rr} \end{array}\right]$$

(16) $$M_{Diag}^{\left(i,t\right)}=\left[\begin{array}{cc} F_{fl}+F_{rr} & F_{fl}+F_{rr}\\ F_{rl}+F_{fr} & F_{rl}+F_{fr} \end{array}\right]$$

where the four distinct matrices $M^{\left(i,t\right)}$, $M_{LR}^{\left(i,t\right)}$, $M_{Front}^{\left(i,t\right)}$, and $M_{Diag}^{\left(i,t\right)}$ correspond to the raw load matrix, left/right load difference matrix, front/rear axle load matrix, and diagonal coupling load matrix of the vehicle wheels, respectively. The variables $F_{fl}$, $F_{fr}$, $F_{rl}$, and $F_{rr}$ denote the vertical forces (normal to the roll table) acting on the front-left, front-right, rear-left, and rear-right wheels, respectively.

**Figure 4:**
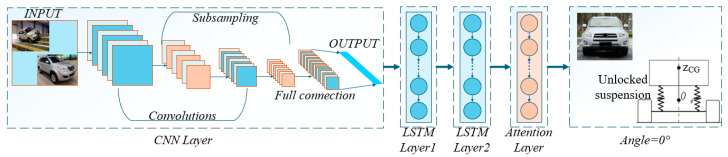
The CNN–LSTM–Attention model.

Although CNNs effectively extract load transfer features during vehicle roll via convolutional and pooling layers, they exhibit temporal insensitivity when processing roll sequence data. Conversely, LSTMs excel at capturing long-term dependencies in roll sequences, addressing the challenge of modeling large roll-angle interval dependencies. Feeding CNN-extracted features into LSTM enables more effective analysis of periodic variation patterns among load transfer, suspension stiffness, and roll angle throughout the roll process. Within nonparametric modeling frameworks, the CNN-LSTM architecture demonstrates nonlinear fitting capabilities; however, its hyperparameter optimization often relies on experience-driven trial-and-error, directly impacting training efficacy. Thus, integrating an attention mechanism enhances extraction of sequence-specific features, enabling precise focus on critical information to improve predictive performance. Accordingly, the CNN–LSTM–Attention predictive model illustrated in Figure 4 is proposed, and its structure is shown in Table 2.

### 3.5. Prediction Workflow of the CNN–LSTM–Attention Model

**Figure 5:**
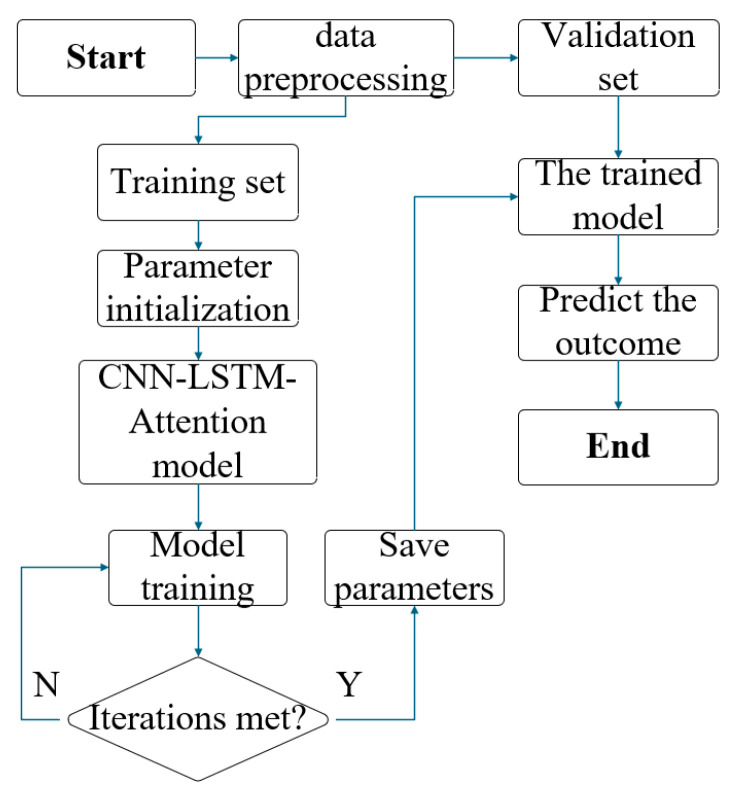
Model prediction process.

Under the test conditions of unlocked suspension and variable sprung mass on a tilt-table platform, *Z_CG_* is predicted. As illustrated in Figure 5, wheel load parameters—including raw wheel loads, lateral load differences, front/rear axle loads, and cross-axle coupling metrics—undergo preprocessing to ensure data quality and format compatibility. The dataset is partitioned into training and testing subsets, with the former used for model training and the latter for predictive performance validation. Prior to model deployment, parameters are initialized. Subsequent training with the dataset optimizes parameters through loss minimization, enhancing prediction accuracy. Convergence status is monitored; training terminates upon convergence, otherwise iterates. Optimized parameters are then archived. The trained model executes inverse temporal predictions (12–0° roll angles) on test data to generate *Z_CG_* estimates.

## 4. Experimental Analysis

### 4.1. Dataset Construction

**Figure 6:**
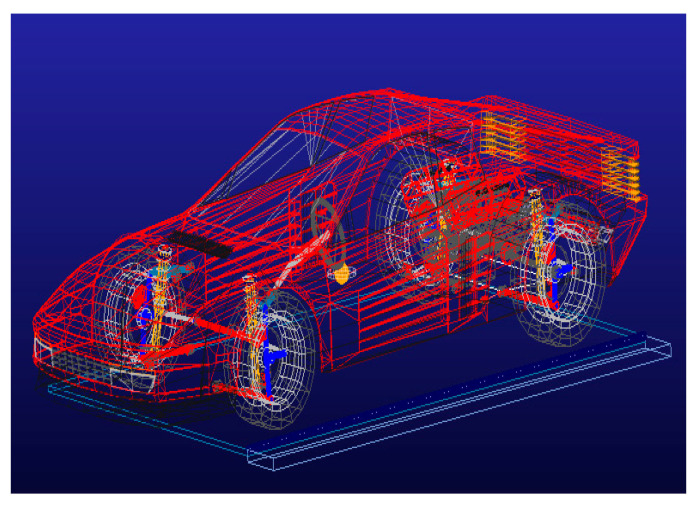
Vehicle theoretical model.

As depicted in Figure 6, a stock vehicle theoretical model with unlocked suspension is established in Adams using the tilt-table method. Suspension stiffness is maintained constant while variable sprung mass conditions are simulated by adjusting component masses. Left/right roll-angle data (0–12°) from the theoretical model comprise the training set for predictive modeling.

As tabulated in Table 3, the test set contains left/right roll-angle data (0–12°) measured on a manufacturer’s tilt-table platform, featuring unlocked suspension and variable sprung mass configurations.

### 4.2. Parameter Configuration and Evaluation Metrics

The computational environment comprised Windows 11 OS; Intel Core i5-14600KF CPU; NVIDIA GeForce RTX 3080 Ti GPU; 32GB RAM; Python 3.11.10; and PyTorch 2.5.1 framework. Model configurations utilized ReLU activation, Adam optimizer (learning rate = 0.001), early stopping with 15-epoch patience, 500 training iterations, and 120 samples per batch.

To evaluate the performance of the proposed model in predicting the *Z_CG_* during tilt-table testing with an unlocked suspension and variable sprung mass, four commonly used metrics were adopted: mean absolute error (*MAE*), mean squared error (*MSE*), mean absolute percentage error (*MAPE*), and root mean squared error (*RMSE*), as defined in Equations (17)–(20) [28]. Lower values of these metrics indicate higher prediction accuracy of the *Z_CG_*.

(17) $$MAE=\frac{\sum_{i}^{m}\left|y_{i}-\tilde{y_{i}}\right|}{m}$$

(18) $$MSE=\frac{1}{m}\sum_{i}^{m}{\left(y_{i}-\bar{y}\right)}^{2}$$

(19) $$MAPE=\frac{1}{m}\sum_{i=1}^{m}\frac{\left|y_{i}-\tilde{y_{i}}\right|}{y_{i}}\times 100\%$$

(20) $$RMSE=\sqrt{\frac{1}{m}\sum_{i=1}^{m}{\left(y_{i}-\tilde{y_{i}}\right)}^{2}}$$

where m is the sample size of vehicle roll-angle data; $y_{i}$ denotes ground-truth *Z_CG_*; $\tilde{y_{i}}$ indicates predicted values; and $\bar{y}$ represents the prediction mean.

### 4.3. Model Prediction and Error Analysis

To validate the *Z_CG_* prediction performance of the CNN–LSTM–Attention model under tilt-table testing conditions with unlocked suspension and variable sprung mass, comparative evaluations were conducted against CNN, CNN-LSTM, and Transformer models. All models utilized identical training and testing datasets.

As quantified by the MAE, MSE, MAPE, and RMSE metrics in Table 4, the CNN–LSTM–Attention model demonstrates superior fitting performance. Across all six prediction trials, this model consistently outperforms its counterparts in all error metrics, indicating optimal predictive capability and implementation feasibility. The CNN-LSTM model exhibits competitive performance with high feasibility, though marginally inferior to CNN–LSTM–Attention in metric evaluations. Conversely, the Transformer model yields moderate performance metrics, signifying intermediate feasibility levels. The CNN model registers the highest error values across all metrics, reflecting the least desirable prediction accuracy and minimal feasibility.

**Figure 7:**
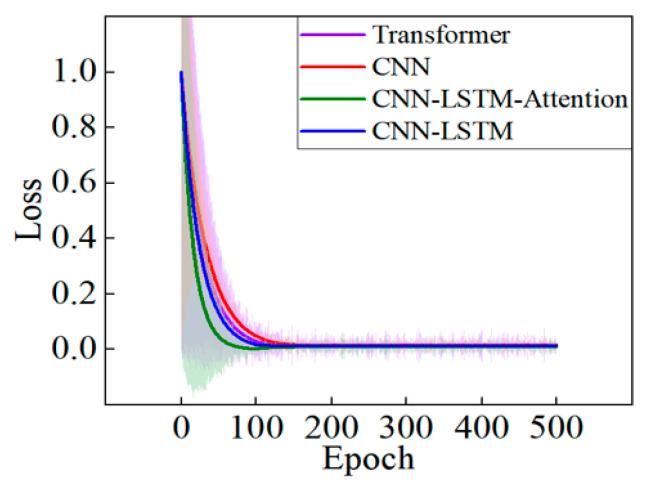
Comparison of training loss curves of four models.

Figure 7 demonstrates decreasing loss trends across all models with increasing epochs, indicating effective optimization. Convergence initiates around epoch 100, where both CNN–LSTM–Attention and Transformer models exhibit faster convergence rates and lower loss values, signifying enhanced fitting capabilities. Post-epoch 100, the CNN–LSTM–Attention model maintains superior stability and minimal loss. The CNN-LSTM model shows slower convergence with marginally inferior performance. In contrast, the standalone CNN model displays the highest initial loss and slowest convergence, indicating its weakest performance in *Z_CG_* prediction.

**Figure 8:**
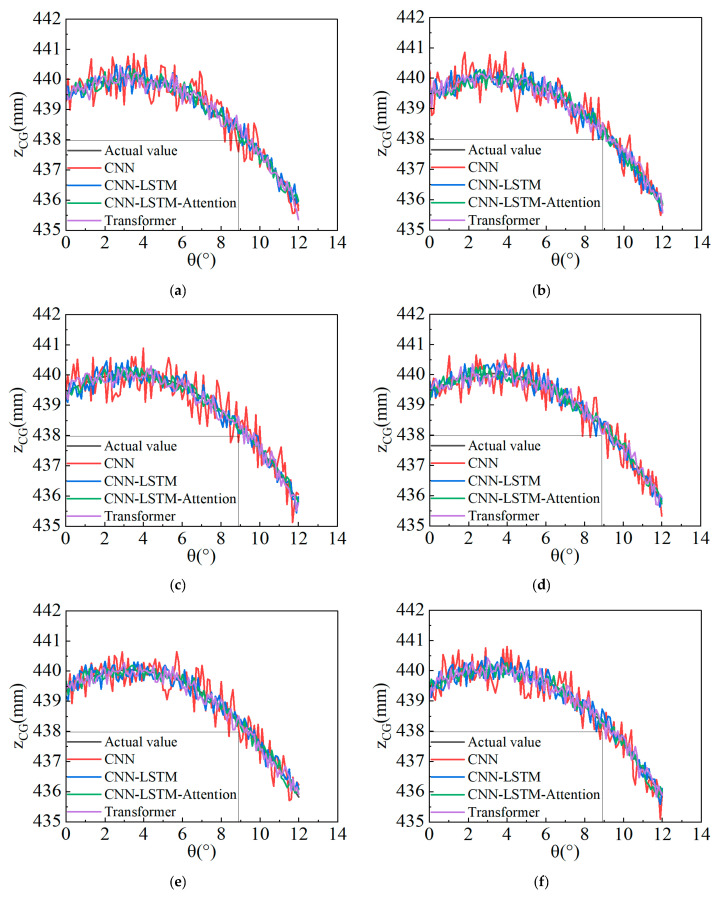
Comparison of prediction results of different models. (**a**) Comparison of the prediction results of each model in the first group with the true values; (**b**) Comparison of the prediction results of each model in the second group with the true values; (**c**) Comparison of the prediction results of each model in the third group with the true values; (**d**) Comparison of the prediction results of each model in the fourth group with the true values; (**e**) Comparison of the prediction results of each model in the fifth group with the true values; (**f**) Comparison of the prediction results of each model in the sixth group with the true values.

Figure 8 compares predicted versus ground-truth *Z_CG_* height at 0° roll angle under tilt-table testing with unlocked suspension and variable sprung mass. Increasing fluctuation amplitudes in the curves correlate with decreasing prediction accuracy. Among six prediction groups, (1) the standalone CNN model yields the poorest performance; (2) Transformer shows marginal improvement but significant residuals; (3) CNN-LSTM surpasses both predecessors; (4) and CNN–LSTM–Attention achieves optimal performance with minimal curve fluctuations in both near 0° (low roll-angle) and near 12° (high roll-angle) regions. The predicted *Z_CG_* values demonstrate the closest alignment with ground-truth data, confirming the superiority of CNN–LSTM–Attention over benchmark models.

As evidenced in Table 5, the CNN–LSTM–Attention model demonstrates superior prediction accuracy and reduced computational time for *Z_CG_* estimation compared to CNN, CNN-LSTM, and Transformer benchmarks.

Table 6 compares 0° *Z_CG_* prediction errors between six CNN–LSTM–Attention implementations and the arithmetic mean method using 6–12° bilateral roll data. Under tilt-table testing with unlocked suspension and variable sprung mass, the CNN–LSTM–Attention model achieves consistently lower errors than the angular averaging approach.

## 5. Conclusions

To mitigate the influence of unlocked suspension and variable sprung mass on *Z_CG_* measurements in tilt-table testing, a CNN–LSTM–Attention prediction model is proposed. Specifically, (1) CNN extracts fine-grained features among load transfer, suspension stiffness, and roll angle during unlocked suspension articulation; (2) LSTM processes temporal dependencies in roll-angle sequences; and (3) the attention mechanism dynamically weights features to accommodate variable sprung mass conditions. Experimental validation on production vehicles demonstrates superior *Z_CG_* prediction accuracy compared to benchmark models, with predictions exhibiting the closest alignment to ground-truth values. This deep learning framework provides a novel solution for vehicle center-of-mass positioning via tilt-table testing, utilizing the CNN–LSTM–Attention model to calculate *Z_CG_* coordinates. The trained model extracts intrinsic relationships among *Z_CG_* position, suspension elastic deformation, wheel-load transfer, and roll angle. It enables accurate *Z_CG_* prediction under unlocked suspension deformation and variable sprung mass scenarios, demonstrating significant practical utility in vehicle statics and dynamics applications.

## Figures and Tables

**Table 1:** The variation of the lateral position *Y_CG_* of the left and right tilting centroids with the tilting angle.

Angle (°)	Left *Y_CG_* (mm)	Right *Y_CG_* (mm)	Average *Y_CG_* (mm)	Error (%)
0	2.32	2.32	2.32	0%
2	25.31	−20.66	2.33	0.22%
4	48.50	−43.85	2.33	0.22%
6	71.98	−67.33	2.33	0.22%
8	95.83	−91.17	2.33	0.43%
10	120.13	−115.46	2.34	0.65%
12	144.96	−140.30	2.33	0.43%

**Table 2:** Structure of the CNN–LSTM–Attention model.

Layer	Output Shape	Core Configuration
Input Layer	(120, 4, 2, 2)	120 timesteps, 4 channels, 2 × 2 matrices
Convolutional Layer 1	(120, 32, 1, 1)	Kernel size: 2 × 2, 32 filters, Stride: 1, Activation: ReLU
Pooling Layer 1	(120, 32, 1, 1)	Max pooling
Convolutional Layer 2	(120, 64, 1, 1)	Kernel size: 2 × 2, 64 filters, Stride: 1, Activation: ReLU
Pooling Layer 2	(120, 64, 1, 1)	Max pooling
Flatten Layer	(120, 64)	Converting spatial features to vectors
LSTM Layer 1	(120, 128)	Unidirectional LSTM, Hidden units: 128
LSTM Layer 2	(120, 64)	Unidirectional LSTM, Hidden units: 64
Attention Layer	(64)	Content-based attention mechanism
Fully Connected Layer	(64)	Activation: ReLU
Output Layer	(1)	Regression prediction

**Table 3:** Partial test set data.

Angle (°)	Ffl (N)	Ffr (N)	Frl (N)	Frr (N)
0	3159.30	3145.10	4345.73	4331.30
0.1	3162.70	3141.67	4349.39	4327.64
0.2	3166.08	3138.20	4353.02	4323.96
…	…	…	…	…
11.8	3426.17	2611.60	4594.19	3722.98
11.9	3427.22	2606.10	4594.68	3716.42
12	3428.25	2600.59	4595.14	3709.84

**Table 4:** The average evaluation indicators of each model in the six groups of experiments.

Model	MAE	MSE	MAPE	RMSE
CNN	0.0937	0.0087	0.0965	0.0627
CNN-LSTM	0.0572	0.0048	0.0241	0.0210
CNN–LSTM–Attention	0.0274	0.0029	0.0216	0.0156
Transformer	0.0624	0.0059	0.0442	0.0311

**Table 5:** Comparative prediction performance and computational efficiency of different models at 0° roll angle.

Model	*Z_CG_* (mm)	Elapsed Time (s)
Actual value	439.44	0
CNN	451.15	101.47
CNN-LSTM	447.75	99.52
CNN–LSTM–Attention	442.64	92.69
Transformer	448.26	104.17

**Table 6:** Comparison of six sets of CNN–LSTM–Attention model prediction results, 6–12° arithmetic average results, and real 0° center of mass height *Z_CG_* error.

Group	CNN–LSTM–Attention (%)	Arithmetic Mean of 6–12°on Both Sides (%)
1	−0.16	−0.31
2	−0.19	−0.29
3	0.27	0.32
4	−0.30	−0.31
5	0.25	0.30
6	0.21	0.31

## Data Availability

Due to the confidentiality requirements of the tested vehicles, the data provided in this study were provided at the request of the corresponding author. Further research on these data will be conducted in the future.

## Abbreviations

The following abbreviations are used in this manuscript:

*X_CG_*	The longitudinal position of the center of gravity
*Y_CG_*	The lateral position of the center of gravity
*Z_CG_*	The height of the center of gravity
CNNs	Convolutional neural networks
LSTMs	Long short-term memory networks
